# Effects of Polyphenol Compounds and Nitrogen Oxide Donors on Lipid Oxidation, Membrane-Skeletal Proteins, and Erythrocyte Structure under Hypoxia

**DOI:** 10.1155/2019/6758017

**Published:** 2019-12-07

**Authors:** Viсtor V. Revin, Natalia V. Gromova, Elvira S. Revina, Ksenia V. Prosnikova, Nadezhda V. Revina, Svetlana S. Bochkareva, Olga G. Stepushkina, Igor P. Grunyushkin, Marina R. Tairova, Vera I. Incina

**Affiliations:** ^1^Department of Biotechnology, Bioengineering and Biochemistry, Faculty of Biotechnology and Biology, Ogarev Mordovian State University, Saransk, Republic of Mordovia 430005, Russia; ^2^Department of Pharmacology and Clinical Pharmacology with a Course of Pharmaceutical Technology, Medicine Institute, Ogarev Mordovian State University, Saransk, Republic of Mordovia 430005, Russia

## Abstract

This study shows that membrane-associated cytoskeletal protein structures and the erythrocyte morphology undergo profound changes during hypoxia. Hypoxia also intensified oxidative processes in the lipid phase of the bilayer of red blood cell membranes. Sodium nitroprusside impaired the morphology of red blood cells and altered quantitative and qualitative composition of membrane-skeletal proteins. The findings suggest that hypoxia causes changes at all levels of red blood cell organization, which can cause the functional disorders of hemoglobin oxygen-transporting properties and, eventually, the complete degradation of red blood cells. The use of flavonoids has a protective effect against hypoxia.

## 1. Introduction

Cardiovascular disease remains one of the most pressing problems in modern medicine. The primary cause is disruption of the structure and function of blood vessel endothelium. However, the role of red blood cells and their oxygen-transport ability in the development of vascular diseases remain poorly understood [1, 2].

Vascular diseases are accompanied by pathological changes in the erythrocytes that disturb the morpho-functional state of erythrocyte membranes and the cell flexibility [3]. It is the spectrin-based cytoskeleton on the cytosolic side of the human red blood cell membrane that confers the mechanical property enabling erythrocytes to withstand the stress on the cell membrane as they are forced through a narrow blood vessel [4–6].

Recent research has shown that nitric oxide is involved in the long-term adaptation of the organism that has strong protective properties during stress situations, including acute hypoxia [7] In this regard, the use of sodium nitroprusside (SN), an exogenous donor of nitric oxide could be of great significance to relieve pathological conditions associated with oxidative stress. It is known that sodium nitroprusside normalizes energy-, nitrogen-, and some other types of metabolism. Its metabolites possess antioxidant effects, suggesting that antioxidant activity could belong to the medicine itself.

In recent years, the researchers' attention was attracted to a group of polyphenolic compounds where flavonoids predominate [8–10]. The flavonoids (FLV) proved their efficiency in decreasing the membrane microviscosity, which plays an important role in maintaining normal erythrocyte deformability, allowing smooth blood flow even through narrow capillaries. Currently, research aims at screening and designing the most suitable flavonoid-based medicines due to their effective prevention of free-radical oxidation of lipids and proteins, which leads to the lysis of erythrocytes [11].

A substance of a polyphenolic nature, resveratrol (RVT) isolated from the dark-skinned grapes and grape seeds is of special interest, as it possess anticarcinogenic, hepatoprotective, anti-inflammatory, and membrane-protective properties [12]. In addition, resveratrol reduces lipid peroxidation and protein oxidation in cell membranes induced by reactive oxygen species. Resveratrol activates also the synthesis of antioxidant enzymes in cells [13].

Considering the above arguments, we considered it challenging to study the interrelation between changes in the structure and composition of membrane-skeletal proteins of red blood cells and the rate of oxidative processes in the lipid bilayer of erythrocyte membranes under hypoxia, in the absence and presence of nitric oxide donors and compounds of polyphenolic nature. That was the main goal of our research.

## 2. Materials and Methods

### 2.1. Experimental Setup

Human blood was taken from healthy persons (blood donors) at the Mordovian Republican Station of Blood Transfusion, Saransk. Donors were men aged 25–45 years (*n* = 56). The study was made possible with the permission of the local ethics committee at Mordovia State University in accordance with the principles of Good Clinical Practice (protocol no. 12 of September 17, 2014). Written informed consent was obtained from each participant of this study.

Blood was taken in the morning on an empty stomach from the cubital vein into vacuum tubes; it was later used to obtain erythrocyte suspensions.

The erythrocytes were divided into two experimental groups. The first group consisted of control sample erythrocytes incubated with the addition of SN and antioxidants, namely quercetin (QC) and resveratrol (RVT). The second group contained the samples incubated under hypoxia in the absence and presence of SN, QC, and RVT.

The experiment scheme is presented in Table 1.

### 2.2. Obtaining Erythrocyte Suspension

Sodium citrate was used as an anticoagulant at a final concentration of 130 mM and pH 7.4. Human erythrocytes were obtained by centrifugation of whole blood at 1000*g* for 10 min. The plasma and leukocyte filming were discarded; the sediment was resuspended in a tenfold volume of erythrocyte incubation medium containing 10 mM KH_2_PO_4_, 3.5 mM KCl, 1.5 mM MgCl_2_, 145 mM NaCl, and 6 mM glucose at pH 7.4. The erythrocytes were washed three times in this medium. The resulting erythrocyte sediment was diluted with the medium to 1 : 5 ratio (*V* : *V*). The experiment was carried out immediately after receiving the red blood cells.

### 2.3. Incubation of Erythrocytes

The erythrocytic mass was well stirred and incubated for 60 min. The thermostated chamber ensured a stable temperature of 37°C.

To create experimental hypoxia, we used a well-known method of passing a constant flow of N_2_ gas through an incubation medium containing the red blood cells [14].

As a basis, we have taken the modified technique described in research work by Wood et al. and Ramser et al. [15, 16].

Incubation of RBCs was carried out in centrifuge tubes hermetically sealed with silicone plugs. We used the equipment developed by the engineers of our department. The N_2_ gas supplemented with 0.04% CO_2_ (PROM-KOMPLEKT-S Trading Company) was carefully passed through the RBC suspension via inlet holes drilled in the silicone tube wall, which fixed thin capillaries (needles) put into the suspension. The gas from tubes was removed through outlet openings. The nitrogen flow rate was 5 cm^3^/min (mL/min). The flushing of N_2_ was carried out at 37°C using a water bath.

The oxygen content in the red blood cell suspension and the time required for complete oxygen displacement were estimated by Raman spectroscopy.

After incubation, erythrocytes were deposited by centrifugation (1500*g*, 10 min), after which the erythrocytic mass was used to obtain membranes.

To study the effects of pro- and antioxidants on erythrocytes under conditions of normoxia and hypoxia, we used SN (as a donor of nitric oxide) and antioxidants (AO): flavonoids (quercetin) extracted from blackcurrants (*Ribes nigrum*) and resveratrol. The final concentration of SN (SNP, Sigma) and RVT (Shaanxi Honghao Bio-Tech Inc., China) in the solution was 100 *μ*M [17–22].

Resveratrol was dissolved in dimethyl sulfoxide (DMSO) to obtain 10 mM solution. This solution was added to the erythrocyte suspension to a final concentration of 100 *μ*M.

### 2.4. Control Treatment of Erythrocytes with DMSO

When the erythrocyte suspension was incubated in the presence of the same amount of DMSO during 60 min, no statistically significant changes were observed in the morphology of erythrocytes, hemoglobin conformation, and antioxidant status (*n* = 56).

### 2.5. Extraction and Separation of Flavonoids from Blackcurrant Fruits

Extraction of flavonoids was performed using an automatic extractor Ase 150 (Deonex, Germany). The sample was prepared as follows: 5 g of crushed fruits was placed in a mortar and mixed with diatomaceous earth at the 1 : 5 ratio. The extraction was performed at 60°C, and a pressure of 1610 PSI (10 PSI = 0.069 MPa) in five cycles was applied. The duration of each cycle was 33 min. The solvent used was 70% ethanol. The prepared extract was filtered through a vacuum membrane filter [23–26].

Separation of flavonoids was performed by thin-layer chromatography on a thin layer of sorbent. Coumarins interfering with the qualitative determination of flavonoids were separated by chloroform. A system of solvents: chloroform-methanol (8 : 3) was used. Identification was carried out by values of the retardation factor (Rf) for individual spots and their staining in UV and visible light [23, 24].

Among the flavonoids isolated from the fruits of blackcurrant, quercetin predominated; this fraction was used in further experiments. Quercetin concentration in the sample was 0.025 mg/ml [25–28].

### 2.6. Raman Spectroscopy

The bands in the Raman spectrum of hemoglobin (Hb) provide information on the Hb conformational structure, its functional condition, and the potential capabilities of Hb at the molecular level. Raman spectroscopy reports on the state of the iron atom and its ligands based on the changes in the structure of the tetrapyrrolic ring of hemoporphyrin [29, 30].

Raman spectra were recorded by means of an inVia Basis Raman spectrometer (Renishaw Company, UK), with a short-focus high-aperture monochromator (focal length ≤ 250 mm). Raman spectra were excited with a laser (532 nm, 100 mW maximum radiation power, 100x lens). Data were recorded by a CCD camera (1024 × 256 pixels) equipped with a Peltier element enabling cooling down to −70°C; grating, 1800 lines/mm.

Spectra were digitized and mathematically processed (correction of baseline and smoothing of Raman spectra) using OriginPro 2015 software [31–34].

When studying hemoglobin conformation, the suspension of red blood cells was put into sealed capillaries of 1 mm in diameter, which were placed in the spectrometer holder. The spectral bands of hemoglobin excited at 532 nm and correlated with porphyrin bond vibrations were recorded. Measurements were performed three times per sample, and the obtained values were averaged.

In preliminary experiments, we used Raman spectroscopy to control hypoxic conditions created in the experiment by analyzing the 1355 and 1375 cm^−1^ bands of the erythrocyte spectrum [35, 36].

**Figure 1:**
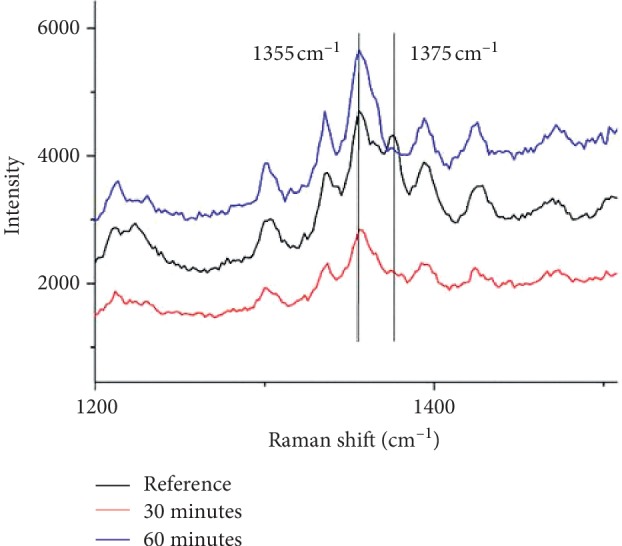
Raman spectra of human erythrocytes after 30-min and 60-min incubation in a gas mixture wherein oxygen was replaced with nitrogen. The control sample was incubated in air for 60 min.

The intensity of spectrum bands at 1355 and 1375 cm^−1^ is linked with symmetrical vibrations of pyrrole rings in the molecule of deoxyhemoglobin and hemoglobin-related ligands, respectively [37]. As the amount of O_2_ in the blood is 3-4 orders higher than the content of other ligands (e.g., NO or CO), the intensity of the 1375 cm^−1^ band is determined mainly by the content of oxyhemoglobin. We found that both 30-min and 60-min oxygen depletion by the method described above resulted in the complete disappearance of oxyhemoglobin, which was proved by the absence of the 1375 cm^−1^ band in the Raman spectrum (Figure 1). This is also proved by the increased peak at 1355 cm^−1^ after 60-min incubation of red blood cells in hypoxic conditions. These facts confirm that the erythrocytes incubated in an oxygen-free environment were actually in a state of hypoxia [38].

### 2.7. Obtaining the Erythrocyte Membranes

The resulting sediment of human erythrocytes was lysed by a 50-fold volume of cold sodium phosphate buffer (5 mM NaH_2_PO_4_ and 0.5 mM PMSF (protease inhibitor), pH 8.0, 4°C). After 15 min, erythrocytes were centrifuged at 18 400*g* for 50 min. The supernatant was discharged. Na_2_HPO_4_ solution was added to the sediment, resuspended, and centrifuged at 18 400*g* for 50 min. The cycle was repeated at least three times. The sediment was washed with a lysing buffer to obtain white membranes. The membranes were stored at −70°C [39].

### 2.8. Measuring Protein Concentration

The protein concentration in the resulting samples was measured with the Lowry method, using bovine serum albumin as a standard [40].

### 2.9. SDS-PAGE

Polyacrylamide gel electrophoresis (PAGE) was performed by the Laemmli method [41]. This method is very effective in determining the protein size [42].

SDS-PAGE was performed on the device, BioRad, using 4% stacking and 10% running gels. Samples were boiled for 5 min in the sample buffer containing 5% *β*-mercaptoethanol at the ratio of 1 : 2. We put 15 *μ*l of the mixture (1 *μ*g protein) in each well. To determine the optimal protein content of mixture loaded per well, precalibration (12.5, 6.25, 3.125, 1.56, and 0.78 *μ*g of protein) was performed. Electrophoresis was performed at a constant current strength (15 and 30 mA on two plates for the movement in stacking and running gels, respectively).

Thereafter, the electrophoregrams were stained for one hour with the Coomassie G-250 dye, according to a modified Fairbanks method in a solution containing 10% acetic acid, 25% isopropanol, and 0.05% Coomassie blue. The unbound dye was washed for 12 h with 10% acetic acid until the background staining completely disappeared [43].

Determination of molecular weight of the studied proteins requires gel calibration of molecular masses. Gels were calibrated with respect to molecular masses of protein markers that were resolved in parallel with the analyzed sample. Mixtures of marker proteins are available for different mass ranges. The molecular masses of the studied proteins were calculated from their electrophoretic mobilities, using regression analysis. Identification of erythrocyte membrane proteins was carried out according to the Fairbanks–Steck classification [43].

Visualization, documentation, and quantitative analysis of the obtained protein gels were made using a gel documenting system Gel Doc XR Plus (Bio-Rad, USA). The results were processed using the Image Lab software.

### 2.10. Atomic Force Microscopy

We used atomic force microscopy (AFM) to study surface structure and morphology of the erythrocytes.

Because of its unique sensitivity, AFM allows measurements in all media, including liquid or physiological environments. This opens the possibility of applying this technique in biology because scanning the sample surface with the tip of the probe located at the end of the cantilever enables the acquisition of 3D images for the morphology of biological samples in their natural condition. During the scanning, the interaction of the tip and the sample causes the deformation of the cantilever. The sensor regulates, collects, and processes the data and actuates piezoelectric scanning to create 3D topography of the scanned area [44, 45].

Three-dimensional images and a cross-sectional profile of nucleus-free erythrocytes were obtained using a scanning probe microscope SPM 9600 (Shimadzu, Kyoto, Japan). The contact mode of scanning was used. This mode allows visualization of cell membrane topography without substantial distortion and deformation. The light source used was a laser diode (650 nm, 1 mW max. power). The scanned area of the sample was 100 × 100 *μ*m, 50 × 50 *μ*m, or 25 × 25 *μ*m, and scanning speed up to 1 Hz. Ten erythrocytes were chosen randomly for each sample, and their profiles were measured in two regions (points). The data were processed using the attached software “SPM Manager Version 3.04,” which determines the cell diameter and cell height [46].

To evaluate the quality of red blood cells, cytological preparations of erythrocyte-containing samples were set down by air-drying, as this procedure does not change the erythrocyte shape or membrane structure. The scanning in the air medium allows to record the minimum distortion of the membrane structure, whereas the spatial resolution in a liquid medium is highly limited [47].

#### 2.10.1. Assay of Diene Conjugates (DC) in Erythrocytes by Placer's Method [48] as Modified by Vladimirov et al. [49] and Gavrilov et al. [50]

The concentration of primary products of lipid peroxidation (LPO), diene conjugates (DC) in erythrocytes, was measured by the spectrophotometric method [48–50].

The method of DC content determination in red blood cells is based on light absorption by lipid extracts in the UV spectral region since molecules with two conjugated bonds have a maximum absorption at 232–233 nm.

Half a milliliter of erythrocyte hemolysate was mixed in test tubes with 4 mL of the isopropanol-heptane mixture (1 : 1, v/v). The mixture was periodically shaken for at least one hour. Then, 1 ml of 0.01 N HCl (pH 2) was added to the mixture and shaken for 2 min.; Thereafter, 2 ml of pure distilled heptane was added and again periodically shaken for 10–15 min. Following 1- or 2-h incubation, the upper phase was collected and taken for the photometric scanning at 232 nm with regard to the control sample (*A*_cs_), where 0.5 ml H_2_O was present instead of hemolysate.

### 2.11. Formula for Calculation

(1) $$\begin{array}{c} C=\left(A_{\text{od}}-A_{\text{cs}}\right)\times 36.4, \end{array}$$

where *A*_od_ is the optical density of the test sample, *A*_cs is the_ optical density of the control sample, and 36.4 is the conversion factor in *μ*mol/l.

### 2.12. Estimation of TBARS (Thiobarbituric Acid Reactive Substances) Concentration by the Method of Ohkawa et al. [51]

The content of the final product of lipid peroxidation, malondialdehyde (MDA), was determined by the reaction with thiobarbituric acid (TBA) [51].

The method assumes that at high temperature in an acidic medium, MDA reacts with TBA, forming a stained trimethine complex with a maximum absorption at 535 nm.

Analyzed sample: 0.1 ml of erythrocyte lysate diluted 18 times

  0.2 ml of 8.1% sodium dodecyl sulfate (SDS)
  1.5 ml of 20% СН_3_СООNa (pH 3.4)
  1.5 ml of 0.8% TBA
  0.7 ml of H_2_O

Blank sample 1 (reference sample without erythrocyte lysate): 0.1 ml of saline solution

  0.2 ml 8.1% SDS
  1.5 ml of 20% СН_3_СООNa (pH 3.4)
  1.5 ml of 0.8% TBA
  0.7 ml of H_2_O

Blank sample 2 (no TBA): 0.1 ml of red blood cell lysate diluted 18 times

  0.2 ml of 8.1% SDS
  1.5 ml of 20% СН_3_СООNa (pH = 3.4)
  2.2 ml of H_2_O

The analyzed and blank samples with a total volume of 4 mL each were boiled for 1 hour (the test tubes were closed with glass stoppers to retain the content at a constant volume). The samples were cooled and centrifuged for 10 min at 4000*g*. Absorbance was measured at a wavelength of 535 nm.

The amount of MDA in erythrocytes was calculated with the following formula:

(2) $$\begin{array}{c} \left[C\right]=\Delta A\times \frac{V}{\varepsilon }\times \frac{d}{\upsilon }=\frac{\Delta A}{\varepsilon }\times d\times \varphi , \end{array}$$

where *φ* = *V*/*υ*, Δ*А* = sample − (blank 1 + blank 2), *ε* is the coefficient of molar extinction of the stained TBA-MDA complex at 535 nm equal to 1.56 × 10^5^ M^−1^×cm^−1^, *d* is the optical path length (1 cm), *V*is the sample volume (4 ml), and *υ* is the the volume of red blood cells in the mixture (0.1 ml).

If the diluted sample of red blood cells is used, the resulting value should be multiplied by the dilution coefficient. Taking into account the conversion of units, the formula is as follows:

(3) $$\begin{array}{c} \left[C\right]=\left(1000\times \Delta A\times \frac{\varphi }{\varepsilon }\right)\times X, \end{array}$$

where 1000 is the conversion factor in *μ*mol/l and *X* is the red blood cell dilution ratio.

### 2.13. Data Analysis

Statistical analysis was carried out using the program of Statistics 6.0. We first assessed the normality of value distributions for each of the samples using Geary's criterion [52]. The homogeneity of dispersion was then evaluated, and ANOVA for repeated measurements was performed. In the case of statistically significant differences between the average values, Tukey's method was used to determine the confidence intervals [53].

The results are presented as arithmetical means and standard deviations (means ± SD).

## 3. Results

### 3.1. Study of Surface Structure and Morphology of Erythrocytes by AFM

The erythrocyte deformability enabling oxygen transport is directly related to morphometric characteristics of cells, the cell shape in particular. Atomic force microscopy is the most effective method to explore morpho-functional changes of the surface and shape of a biological object without causing destruction under air or liquid exposure.

Considering the disc shape of red blood cells, two morphometric parameters were chosen to examine its changes: the diameter and thickness. Both parameters can be reliably determined during AFM scanning of the cytological preparations.

**Figure 2:**
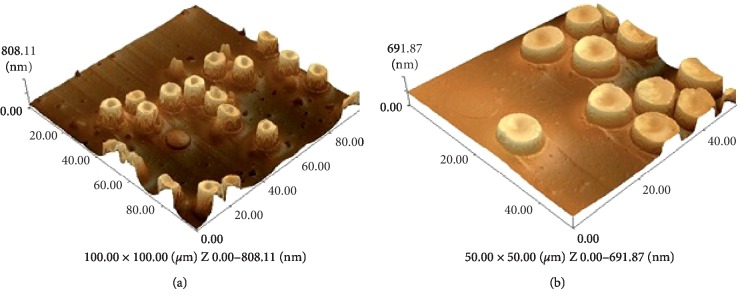
AFM surface images of human erythrocyte test samples: (a) 100 × 100 *μ*m and (b) 50 × 50 *μ*m.

When scanning a control sample of erythrocytes, we obtained images with a shape typical of erythrocytes, a toroidal discocyte. According to the cross-sectional profiles obtained by scanning in the AFM contact mode, the average diameter of cells was 8.27 ± 0.34 *μ*m, which corresponds to a diameter range of normal red blood cells. The mean thickness of erythrocytes amounted to 0.810 ± 0.017 *μ*m (Figures 2(a) and 2(b); Table 2).

After incubation of erythrocytes with sodium nitroprusside, the shape of the main part of erythrocytes in the first series samples is also typical, discoid, while part of the erythrocytes lost their central pallor (planocytes). The average diameter of cells remained within normal parameters and amounted to 8.69 ± 0.41 *μ*m. Erythrocyte thickness decreased by 14% (Table 2).

After incubation with quercetin, AFM images show that the erythrocytes had lost their biconcave shape (planocytes). According to the cross-sectional profile, the average diameter of cells increased by 12.05% to 9.27 ± 0.44 *μ*m. The average erythrocyte thickness decreased by 17%.

This effect is probably related to the properties of certain groups of quercetin. The chemical structure of the quercetin molecule accounts for its apparent antioxidant properties. Due to the large number of hydroxyl groups and conjugated *π*-orbitals, quercetin can act as an electron donor or hydrogen donor, thus binding H_2_O_2_ and oxidizing superoxide anion (anion peroxide). This ensures neutralisation of free radicals, causing the formation of a semiquinone-radical and then H_2_O_2_. Quercetin reacts with H_2_O_2_ in the presence of peroxidase, thus reducing the concentration of hydrogen peroxide and preventing damage to the cells caused by the latter [54].

After incubation with resveratrol, erythrocytes retained their typical shape, with a distinct hollow portion in the middle of the cell.

**Figure 3:**
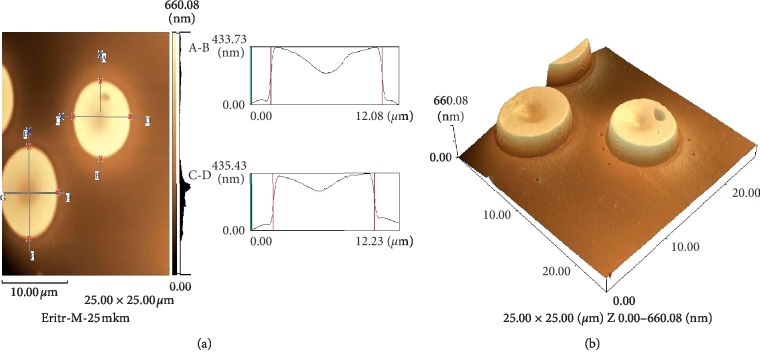
A cross-sectional profile (a) and 3D image (b) of human erythrocytes after exposure to resveratrol (25 × 25 *μ*m).

The average diameter did not change in comparison with the diameter of untreated cells and was 8.15 ± 0.21 *μ*m. The erythrocyte thickness decreased by 13% (Figures 3(a) and 3(b)).

**Figure 4:**
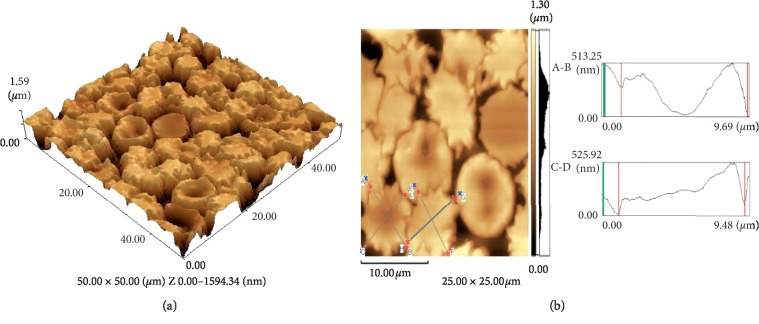
AFM image (a) of the surface of human erythrocytes during hypoxia (50 × 50 *μ*m) and (b) a cross-sectional profile of human erythrocytes during hypoxia (25 × 25 *μ*m).

After the hypoxic treatment, erythrocytes change their shapes from toroidal discocytes to echinocytes and stomatocytes. The cross-sectional profile of erythrocytes shows a significant increase in the roughness of its surface, especially at the periphery. The average diameter of cells has decreased by 20.72% (6.7 ± 0.20 *μ*m). The average erythrocyte thickness increased by 39% (Figures 4(a) and 4(b)).

After erythrocytes were incubated with SN during hypoxia, the cell shape remodels from typical biconcave discocytes into transitional forms resembling echinocytes. The roughness of the membrane at the cell periphery significantly increased. The average diameter and cell thickness increased by 23.66% and 26%, respectively.

**Figure 5:**
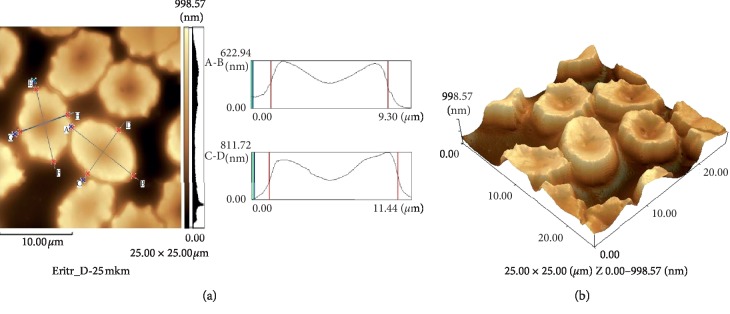
Profile (a) and 3D image (b) of human erythrocytes after exposure to quercetin during hypoxia (25 × 25 *μ*m).

After incubation of erythrocytes with quercetin during hypoxia, we noticed the roughness of the peripheral cellular structure as well as of the cell surface. However, as one can see in the cross-sectional profile of the erythrocyte, we clearly observed a hollow indentation in the middle of the cell. The average diameter and cells' thickness increased by 6.65% and 13%, respectively (Figures 5(a) and 5(b)).

**Figure 6:**
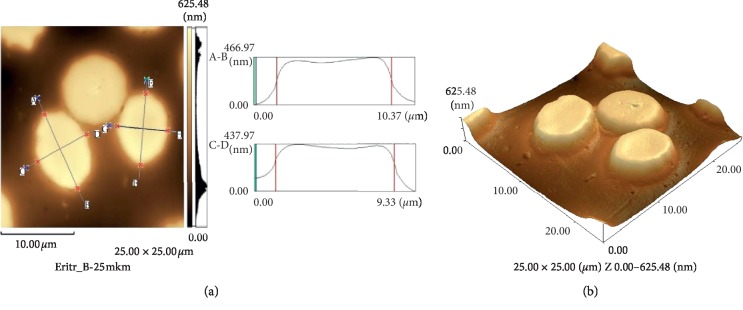
Profile (a) and 3D image (b) of human erythrocytes after exposure to resveratrol under hypoxia (25 × 25 *μ*m).

After erythrocytes were incubated with resveratrol during hypoxia, we mostly obtained the images of discocytes (Figures 6(a) and 6(b)).

However, according to the profile cross section, the erythrocytes had small irregularities of the cytoskeleton along the periphery. The average diameter of the cells did not change in comparison with the control group and amounted to 8.38 ± 0.31 *μ*m. The value of erythrocyte thickness remained within normal limits.

Thus, we have found significant changes occur in the structure of red blood cells during hypoxia. The morphology of erythrocytes is sensitive to the action of sodium nitroprusside and phenolic compounds of natural origin. Considering the abundant literature [55–57] and the results of our previous study of the lipid component of erythrocyte membranes [58, 59], we thought that the structure and condition of erythrocytes is interconnected not only with the lipid components of membranes, but also with proteins of the cytoskeleton. Therefore, in the second series of experiments, we quantified the condition of the erythrocyte membrane-skeletal proteins during hypoxia and treatment with nitroprusside sodium and quercetin.

Therefore, in the control sample, we observed mainly discocytes. After incubation with SN, quercetin, and resveratrol, RBC thickness decreased slightly, whereas quercetin caused the increase in cell diameter. SN and quercetin stimulated the formation of planocytes in the samples, and resveratrol contributed to the preservation of the typical RBC toroidal form with pellor. Hypoxia revealed the increased proportion of echinocytes and stomatocytes in the samples, while the RBC diameter decreased and the thickness increased.

Incubation under hypoxia with SN and quercetin led to the increase in the proportion of transitional and echinocytic forms in the samples, while the RBC diameter and thickness increased. Resveratrol in hypoxia had an evident protective effect, the samples were dominated by discocytes, and the RBC diameter and thickness did not differ from the control samples.

### 3.2. Quantitative Analysis of Membrane-Skeletal Proteins

The condition of erythrocyte membrane-skeletal proteins was quantified by the Lowry method.

When erythrocytes are incubated under normoxia in the presence of SN, QC, and RVT, no significant changes in the protein content of erythrocyte samples were observed.

**Figure 7:**
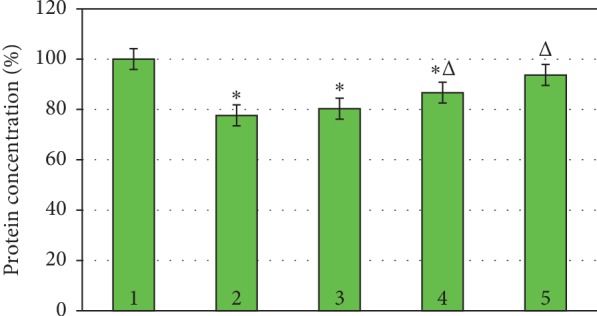
Concentration of membrane-skeletal proteins in the experimental samples of human erythrocytes during hypoxia. ^*∗*^*P* ≤ 0.05 as related to the control; ^Δ^*P* ≤ 0.05 as related to the previous degree of hypoxia. 1, control normoxia; 2, hypoxia; 3, hypoxia + SN; 4, hypoxia + QC; 5, hypoxia + RVT.

Incubation of erythrocytes in hypoxic conditions decreased the concentration of membrane and cytoskeletal proteins in comparison with the control value by 22.36% (Figure 7).

When NP was added to the erythrocyte incubation medium during hypoxia, the concentration of protein in the samples decreased by 19.71% in comparison with the control values. When flavonoids and resveratrol were added to the incubation medium, we observed a decrease in protein concentration by 13.35% and 6.30%, respectively (Figure 7).

Therefore, after exposure to SN, FL, and RVT in normoxia, the protein content in the samples does not differ from the control samples. Incubation of erythrocytes in hypoxia exposed to SN, FL, and RVT was accompanied by the decrease in the concentration of separate fractions (studied protein fractions). It should be underscored that after exposure to resveratrol, changes in the proteins composition were less evident.

### 3.3. Electrophoretic Analysis of Erythrocytes Membrane-Skeletal Proteins

Changes in the shape of erythrocytes could be caused by several factors. First of all, the disturbance in the composition of phospholipids in the lipid bilayer of erythrocyte membranes may affect the membrane microviscosity. Apart from that the imbalance of the membrane-surface charges contributes to additional interactions between proteins and lipids, which ultimately modify the entire cell cytoskeleton [60, 61].

Most likely, a combination of these processes plays a significant part in changes of the erythrocyte morphology.

**Figure 8:**
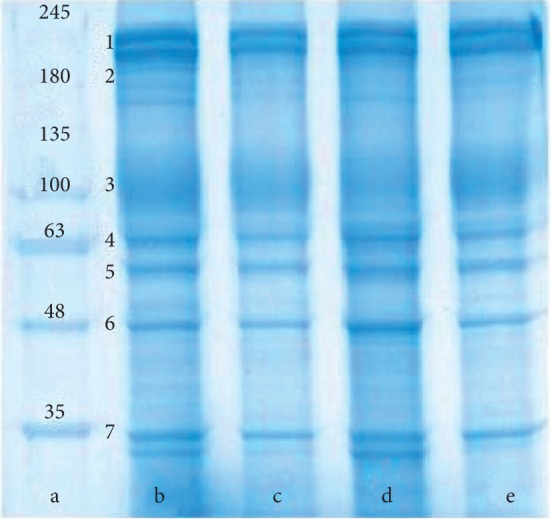
Electrophoregram of cytoskeletal proteins of human erythrocytes (normoxia): a, marker; b, control; c, QC; d, RVT; e, NP. 1, spectrin; 2, ankyrin; 3, band 3 protein; 4, band 4.1 protein; 5, band 4.2 protein; 6, actin; 7, GAPDH.

By applying electrophoresis, we isolated the following main membrane proteins of erythrocytes: spectrin, ankyrin, band 4.1 protein, band 4.2 protein, and actin (proteins that form the membrane skeleton of the erythrocyte), as well as the band 3 protein and glyceraldehyde-3-phosphate dehydrogenase (GAPDH) (proteins involved in metabolism and ion homeostasis of erythrocyte) [62] (Figure 8).

The presence of sodium nitroprusside in the incubation medium changed the appearance of the electrophoregram. The amount of spectrin and actin decreased (by 22.0% and 11.8%, respectively). The amount of ankyrin and band 3 protein decreased almost twofold. The contents of GAPDH remained unchanged. There were no bands corresponding to band 4.1 protein and band 4.2 protein (Figure 8).

Changes in the protein migration pattern were also observed when quercetin was added to the incubation medium. The amounts of spectrin and band 3 protein decreased significantly (26.0% and 30.0%, respectively). The content of proteins such as ankyrin, band 4.1 protein, and GAPDH decreased to a lesser extent (by 17.9%, 11.6%, and 15.5%, respectively). The content of actin and band 4.2 protein remained unchanged.

The protein pattern of erythrocyte membranes also changed in the resveratrol treatment. There was a noticeable decrease in actin content (by 37.4%) and spectrin content (by 23%). Also, the amount of ankyrin, band 3 protein, band 4.2 protein, and GAPDH decreased in comparison with the control samples (by 12.7%, 15.1%, 6.4%, and 6.8%, respectively). The content of band 4.1 protein remained unchanged.

**Figure 9:**
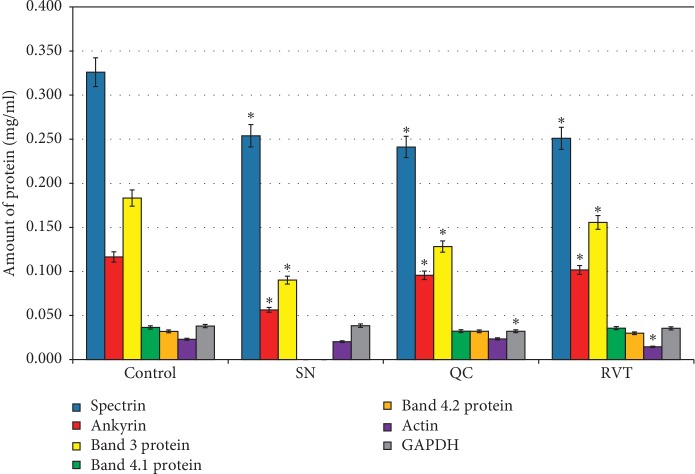
Quantitative composition of erythrocyte proteins in conditions of normoxia (mg/ml). ^*∗*^*P* ≤ 0.05 as related to the control.

Figure 9 represents the quantitative changes in protein composition.

**Figure 10:**
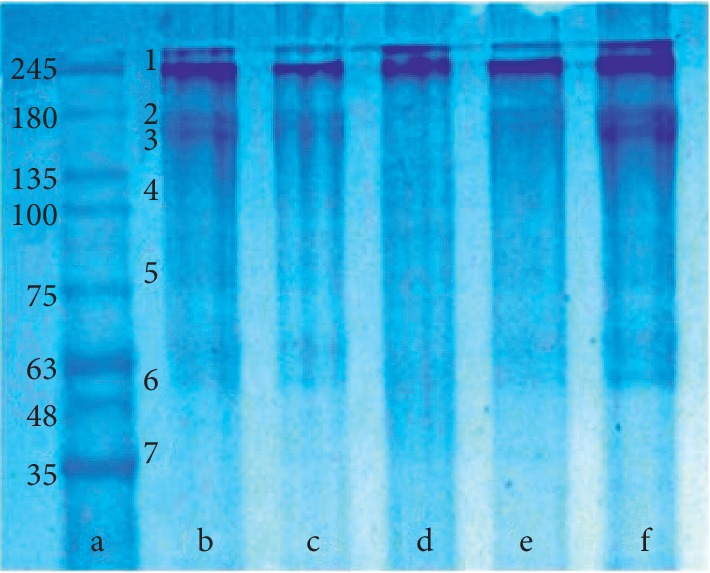
Electrophoregram of cytoskeleton proteins of human erythrocytes exposed to hypoxia: a, marker; b, control; c, hypoxia; d, hypoxia + RVT; e, hypoxia + QC; f, hypoxia + SN. 1, spectrin; 2, ankyrin; 3, band 3 protein; 4, band 4.1 protein; 5, band 4.2 protein; 6, actin; 7, GAPDH.

**Figure 11:**
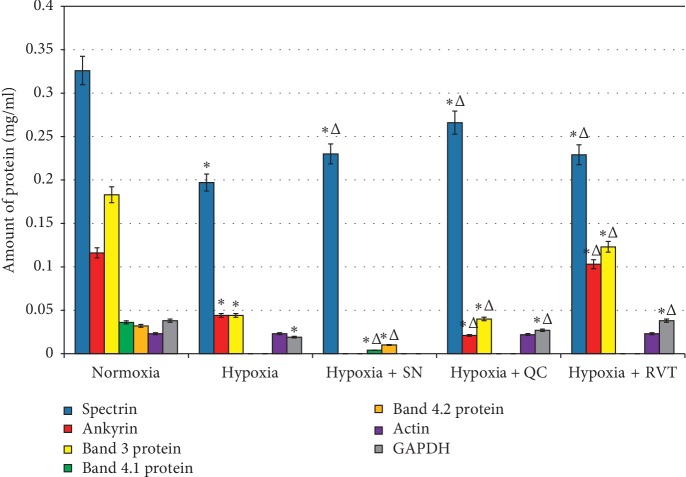
Quantitative composition of erythrocyte proteins in conditions of hypoxia (mg/ml). ^*∗*^*P* ≤ 0.05 as related to the control; ^Δ^*P* ≤ 0.05 as related to the previous degree of hypoxia.

Under hypoxia, there were significant changes in the quantitative composition of protein fractions. The intensities of spectrin, ankyrin, band 3 protein, and GAPDH bands decreased by 39.6%, 62.2%, 76.0%, and 50.0%, respectively. The content of actin did not change. We were unable to detect the bands corresponding to band 4.1 protein and band 4.2 protein (Figures 10 and 11).

When nitroprusside was added under hypoxic conditions, there was a decrease in the amount of spectrin. Band 4.1 protein and band 4.2 protein were observed; however, the content of these proteins was lower than that seen in normoxia. Ankyrin, band 3 protein, actin, and GAPDH completely disappeared under hypoxic conditions.

With quercetin added to the medium under hypoxia, only the content of actin remained almost unaffected by oxygen concentration. Compared with the conditions of hypoxia, the amount of spectrin and GAPDH increased. However, the amount of these proteins is lower than in conditions of normoxia. Band 4.1 protein and band 4.2 protein were missing on the electrophoregram (Figure 10).

With resveratrol in the medium under conditions of hypoxia, the content of spectrin, ankyrin, band 3 protein, and GAPDH increased, as opposed to changes of these proteins under hypoxia without additives. In this case, only the amount of actin and GAPDH corresponded to the level of the control sample. Like in the previous series of experiment, the electrophoregrams revealed neither band 4.1 protein, nor band 4.2 protein (Figures 10 and 11).

Therefore, in normoxia after exposure to SN, the amount of spectrin and actin, ankyrin, and band 3 protein decreased; band 4.1 protein and band 4.2 protein was not present; and the amount of GAPDH did not change. When exposed to quercetin, the content of spectrin, band 3 protein, ankyrin, band 4.1 protein, and GAPDH decreased and the content of actin and band 4.2 protein did not change. Resveratrol led to the decreased content of actin and spectrin, ankyrin, band 3 protein, band 4.2 protein, and GAPDH, and the content of band 4.1 protein did not change. In hypoxia the amount of spectrin, ankyrin, band 3 protein, GAPDH decreased, band 4.1 protein, and band 4.2 protein was not present.

When exposed to SN in hypoxia, there is an increased amount of spectrin, band 4.1 protein, and band 4.2 protein; ankyrin, band 3 protein, actin, and GAPDH were not present. After exposure to flavonoids in hypoxia, the amount of spectrin and GAPDH increased, band 4.1 protein and band 4.2 protein were not present, and band 3 protein and ankyrin decreased. After exposure to resveratrol in hypoxia, the amount of spectrin, ankyrin, band 3 protein, and GAPDH increased and there were no proteins: band 4.1 protein and band 4.2 protein. The content of actin does not change in all samples and corresponds to the values in normoxia.

### 3.4. Analysis of Lipid Peroxidation Rates in Human Erythrocytes

**Figure 12:**
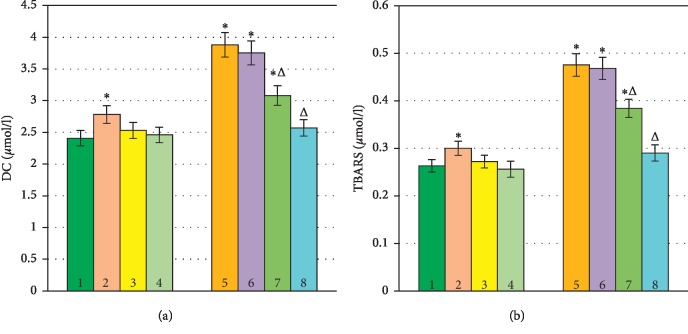
Content of DC (a) and TBARS (b) in human erythrocytes during hypoxia. ^*∗*^*P* ≤ 0.05 as related to the control;^Δ^*P* ≤ 0.05 as related to the previous degree of hypoxia. 1, control normoxia; 2, control + SN; 3, control + QC; 4, control + RVT; 5, hypoxia; 6, hypoxia + SN; 7, hypoxia + QC; 8, hypoxia + RVT.

Considering the changes detected in the composition of proteins and morphology of erythrocytes during hypoxia, we supposed that they can be attributed to the lipid bilayer condition, namely, the oxidative processes in the fatty acid moiety of phospholipids. In order to verify the existence of this mechanism, we conducted a series of experiments in which the intensity of lipid peroxidation was evaluated. For this purpose, we determined the level of accumulation of primary and secondary products of LPO: diene conjugates (DC) and malondialdehyde (MDA). The content of DC in the control sample of human erythrocytes amounted to 2.407 ± 0.181 *μ*mol/l, and the MDA content was 0.263 ± 0.012 *μ*mol/l (Figures 12(a) and 12(b)).

The presence of NP in the incubation medium led to an increase in the amounts of DC and TBARS in erythrocytes by 15% and 14%, respectively (*P* ≤ 0.05). In the case when FL and RVT were added to red blood cells, the content of products of lipid peroxidation was not significantly changed (Figures 12(a) and 12(b)).

During hypoxia, there was a significant increase in the amount of LPO products: DC by 61.6% and MDA by 80.6% compared to normoxic samples.

In the presence of NP, the amount of DC remained almost the same as in the sample with hypoxic conditions (*P* ≤ 0.05). During incubation in similar conditions, but with the additional presence of QC (FL) and RVT, the amount of DC decreased; moreover, in the case of RVT treatment, the content of DC decreased almost to the level of normoxia (Figure 12(a)).

The amount of TBARS in hypoxic conditions varied similarly (Figure 12(b)). The presence of antioxidants in the incubation medium reduced the content of TBARS, but the control level was never achieved.

Therefore, in normoxia, the amount of DC and TBARS increased after exposure to SN. Under hypoxia, as well as after exposure to SN and quercetin, the concentration of DC and TBARS remained above the control values. RVT reduced/neutralised the process of oxidative stress in normoxia and hypoxia.

## 4. Discussion

The erythrocyte membrane is known to account for 1% of the total cell weight. It has high capacity of plasticity and flexibility and contains a number of membrane-bound enzymes required for glycolysis, the pentose phosphate cycle, ion-transport, and glutathione systems, as well as membrane-receptor complexes and antioxidants [63].

Most erythrocyte proteins are localized to certain areas on the inner (cytoplasmic) side of the membrane and form a network of filaments (actin and intermediate filaments) over the cell volume. This network forms a cytoskeleton, which is necessary to maintain the normal shape of the erythrocyte.

Actin microfilaments are composed of two twisted spiral chains of monomeric actin, about 7 nm in diameter and concentrated at the cell membrane. Intermediate filaments consist of subunits with a diameter of 8–11 nm. The constituent proteins include spectrin and glycophorin, which act as a receptor protein [63].

There is a distinction between the integral and peripheral cytoskeleton proteins [5]. The main integral cytoskeleton proteins are band 3 protein and glycophorins A, B, and C. A network of membrane-associated cytoskeleton proteins is composed of spectrin and of short actin filaments, consisting of 12–16 actin monomers.

The attachment of the cytoskeleton to the membrane is achieved by vertical interactions of two types. The interaction of the first type is mediated by ankyrin that links the integral band 3 protein to the site in the middle of the spectrin molecule. This type of interaction also involves the band 4.2 protein.

The second type is mediated by the band 4.1 protein that connects the integral protein glycophorin C to the terminal part of the spectrin molecule. Therefore, the structural integrity of erythrocyte membranes depends on horizontal linkages within the spectrin network and on vertical links between this network and integral membrane proteins [64, 65].

Under the influence of adverse factors, such as hypo- and hyperoxia, the protein structure undergoes alterations, the normal functioning is disturbed, and pathological forms of erythrocytes develop, namely, echinocytes, stomatocytes, and spherocytes, which are impaired in their basic function of oxygen transportation.

Our findings show that incubation of erythrocytes under hypoxia is detrimental to the membrane-skeletal proteins. One factor that diminishes the content of membrane-skeletal proteins is the increased formation of reactive oxygen species, which not only boost the oxidative processes in the lipid phase, but also cause damage to proteins [66]. This is evident from our findings on the accumulation of LPO products in erythrocytes during hypoxia. As a result of protein and lipid damage, the activity of protease and phospholipase intensifies. Protease can break down damaged protein molecules, destroying the sites where cytoskeleton filaments attach to the cell membrane [67].

A detailed analysis of the protein composition by western blotting technique confirms our findings. Under hypoxia, the number of eight protein fractions decreases [68].

The incubation of erythrocytes under hypoxia with sodium nitroprusside also reduced the amount of protein compared to the control samples. This may be attributed to the presence of the cyanide group, CN^−^ in SN, which may have a toxic effect on erythrocytes [41].

A pronounced effect of polyphenols on the quantitative distribution of erythrocyte proteins may be attributed to inactivation of free radicals arising during hypoxia. Moreover, flavonoid substances not only inactivate the free radicals formed but they are also the effective chelators of Fe^2+^ ions known to promote lipid peroxidation [11].

A key step in the action of resveratrol is termination of formation of a resonance-stabilised peroxide radical of RVT. This radical is formed easily due to a high degree of resonance stabilisation. Therefore, energetically, this radical prevails over those which could be converted (produced) from a metahydroxy resorcin group in the other ring [69].

Hypoxia initiated not only quantitative but also qualitative changes in the protein composition of erythrocyte cell membranes. Therefore, the electrophoregrams did not reveal the presence of proteins: band 4.1 protein and band 4.2 protein. At the same time, the intensity of spectrin, ankyrin, band 3 protein, and GAPDH bands reduced significantly. It is likely that the decrease in the number of these proteins was caused by activation of Ca^2+^-dependent proteases [70]. In turn, the inflow of Ca^2+^ ions into erythrocytes may be attributed to the disruption of the regulatory mechanisms of Ca^2+^-mediated ATP-sensitive transport systems because of the intensified peroxidation in the membrane lipid bilayer [70].

In addition, the affinity of hemoglobin to oxygen decreases during hypoxia [71]. The increase of membrane-bound hemoglobin indicated the disruption of the membrane integrity. Furthermore, hemoglobin binds covalently to the membrane skeleton during free-radical oxidation, thus destroying its protein structure and contributing to the oxidation of phospholipids through Ca^2+^-dependent lipo-oxygenase activity. One limiting step for hemolysis is the oxidation of the SH group of membrane proteins by hemin. During this step, denatured hemoglobin reacts easily with the membrane, causing the aggregation of band 3 protein (causing it to bind with its cytoplasmic domain, altering its conformation) and the ultimate destruction of the erythrocyte cytoskeleton [60, 61].

Incubation in presence of sodium nitroprusside and quercetin induced slight qualitative changes in protein composition, which was evident as the decrease in the intensity of protein bands. An electrophoregram of erythrocyte membrane-skeletal proteins incubated in the resveratrol-containing medium did not differ from the control samples.

A stronger protective effect of resveratrol can probably be attributed to structural properties of this substance. According to previous findings, resveratrol scavenges superoxide and hydroxyl radicals, being a donor of H^+^ ions from the hydroxyl groups, whereas OH^−^ groups in positions 3′ and 5′ are the most reactive ones. It was also shown that the 4-hydroxy group in resveratrol was more acidic than the two metha groups, and therefore, any chemical or biological property that heightens resveratrol acidity will probably increase its activity. This effect can be observed when acidosis develops during hypoxia [72].

Changes in the protein composition of erythrocyte membranes and cytoskeleton are accompanied by disturbances of morphological characteristics of erythrocytes, including their diameter and thickness. For instance, the incubation of erythrocytes during experimental hypoxia resulted in significant amounts of red blood cell pathological forms, such as echinocytes and stomatocytes. Perhaps, this was due to partial dehydration and shrinkage of cells after opening of Ca^2+^-dependent K^+^ channels (the Gardos effect), which occurs under the action of oxidants and lipoperoxidation products [73–76].

We suppose that activation of the “Gardos” channels is a common representation of the cell response to oxidative stress. Increasing the level of cytosolic calcium ions, in turn, can intensify the enzyme calpain, which lyses the cytoskeleton network, causing the appearance of bubbles and roughness on the surface of the erythrocyte [77].

Moreover, the reduced levels of ATP during hypoxia contribute to the changes in properties and structure of membranes and cytoskeleton. These changes lead to the transformation of the main population of erythrocytes into the pathological form of the echinocyte that lose their oxygen carrying capacity. This transformation is caused by formation of cross-links between spectrin and hemoglobin [67].

Adding SN and QC to the incubation medium did not contribute to the recovery of the erythrocyte forms produced during hypoxia. This can probably be caused by the fact that in the body, SN functions as a drug precursor: it reacts with sulfhydryl groups of the membrane and plasma, with the release of NO. Nitric oxide stimulates soluble guanylate cyclase to synthesize cGMP, facilitating calcium sequestration and inhibition of cell shrinkage [78].

Resveratrol in the incubation medium contributed to the maintenance of the normal discoidal shape of red blood cells. Perhaps, this was due to the dual antioxidant capacity of resveratrol that initiates the transition of antioxidant enzymes—superoxide dismutase and catalase—into the active state. One mechanism of RVT action is to maintain the level of NADP+, which is used by some antioxidant enzymes for enzyme reduction.

In particular, the level of reduced glutathione (GSH) is maintained through the reduction of the oxidised form (GSSG), which requires NADP^+^ molecules. In addition, for the normal functioning of catalase, NADP^+^ molecules must bind with the catalase tetrameric molecule, ensuring the catalytic decomposition of H_2_O_2_ to O_2_ and H_2_O [79].

It should be noted that the thicknesses of human erythrocytes determined in our study differed from the data found in the literature. This could result from the specific procedures of sample preparation and from the adhesion of red blood cells to the substrate during drying, which predetermined their slight flattening.

## 5. Conclusion

The experiments have shown that hypoxia is a cause of profound changes in the composition of proteins that form the cytoskeleton of human red blood cells. Simultaneously, with changes in the composition of protein fractions, accumulation of fatty acid oxidation products forming a lipid bilayer of erythrocyte membranes was revealed. Intensification of oxidative processes in the bilayer will definitely modify the properties of fatty acids in individual phospholipids, causing phase shift in the bilayer.

Taking into account the whole range of detected changes, we can clearly state that hypoxia disturbs the main function of hemoglobin, oxygen transport. One of the factors that have a protective effect against hypoxia is flavonoid compounds, resveratrol in particular.

## Figures and Tables

**Table 1:** The schematic of experiment.

Experimental conditions	
Control sample	Hypoxia
Control + SN	Hypoxia + SN
Control + QC	Hypoxia + QC
Control + RVT	Hypoxia + RVT

**Table 2:** Morphometric characteristics of erythrocytes during normoxia and hypoxia (mean ± SD).

Experimental conditions	Parametres			
	Normoxia		Hypoxia	
	Diameter (*μ*m)	Thickness (*μ*m)	Diameter (*μ*m)	Thickness (*μ*m)
Control	8.27 ± 0.34	0.810 ± 0.002	6.70 ± 0.20^*∗*^	1.14 ± 0.003^*∗*^^Δ^
+ SN	8.69 ± 0.41	0.697 ± 0.004^*∗*^	8.23 ± 0.09^*∗*^^Δ^	1.20 ± 0.08^*∗*^
+ QC	9.27 ± 0.44^*∗*^	0.672 ± 0.004^*∗*^	8.82 ± 0.1^*∗*^^Δ^	0.915 ± 0.007^*∗*^^Δ^
+ RVT	8.15 ± 0.21	0.705 ± 0.003^*∗*^	8.38 ± 0.31^*∗*^^Δ^	0.809 ± 0.004^*∗*^^Δ^

^*∗*^ *P* ≤ 0.05 as related to the control; ^Δ^*P* ≤ 0.05 as related to the previous degree of hypoxia.

## Data Availability

The data used to support the findings of this study are included within the article, and the original data used to support the findings of this study are available from the corresponding author upon request (Natalia V. Gromova, e-mail: nataly_grom@mail.ru).
